# Online Advertising to Reach and Recruit Latino Smokers to an Internet Cessation Program: Impact and Costs

**DOI:** 10.2196/jmir.2162

**Published:** 2012-08-27

**Authors:** Amanda L Graham, Ye Fang, Jose L Moreno, Shawn L Streiff, Jorge Villegas, Ricardo F Muñoz, Kenneth P Tercyak, Jeanne S Mandelblatt, Donna M Vallone

**Affiliations:** ^1^Schroeder Institute for Tobacco Research & Policy StudiesAmerican Legacy FoundationWashington, DCUnited States; ^2^Department of OncologyGeorgetown University Medical Center/ Cancer Prevention and Control ProgramLombardi Comprehensive Cancer CenterWashington, DCUnited States; ^3^Marketing DepartmentAmerican Legacy FoundationWashington, DCUnited States; ^4^Business AdministrationUniversity of Illinois at SpringfieldSpringfield, ILUnited States; ^5^Department of PsychiatryUniversity of California, San FranciscoSan Francisco, CAUnited States; ^6^Research and Evaluation DepartmentAmerican Legacy FoundationWashington, DCUnited States

**Keywords:** Internet, smoking cessation, recruitment, Hispanic/Latino, advertising

## Abstract

**Background:**

Tobacco cessation among Latinos is a public health priority in the United States, particularly given the relatively high growth of this population segment. Although a substantial percentage of American Latinos use the Internet, they have not engaged in Web-based cessation programs as readily as other racial/ethnic subgroups. A lack of culturally specific advertising efforts may partly explain this disparity.

**Objective:**

Phase I of this study focused on the development of four Spanish-language online banner advertisements to promote a free Spanish-language smoking cessation website (es.BecomeAnEX.org). Phase II examined the relative effectiveness of the four banner ads in reaching and recruiting Latino smokers to the cessation website.

**Methods:**

In Phase I, 200 Spanish-speaking Latino smokers completed an online survey to indicate their preference for Spanish-language banner ads that incorporated either the cultural value of family (*familismo*) or fatalism (*fatalismo*). Ads included variations on message framing (gain vs loss) and depth of cultural targeting (surface vs deep). In Phase II, a Latin square design evaluated the effectiveness of the four preferred ads from Phase I. Ads were systematically rotated across four popular Latino websites (MySpace Latino, MSN Latino, MiGente, and Yahoo! en Español) over four months from August to November 2009. Tracking software recorded ad clicks and registrants on the cessation website. Negative binomial regression and general linear modeling examined the main and interacting effects of message framing and depth of cultural targeting for four outcomes: number of clicks, click-through rate, number of registrants, and cost per registrant.

**Results:**

In Phase I, smokers preferred the four ads featuring *familismo*. In Phase II, 24,829,007 impressions were placed, yielding 24,822 clicks, an overall click-through rate of 0.10%, and 500 registrants (2.77% conversion rate). Advertising costs totaled US $104,669.49, resulting in an overall cost per click of US $4.22 and cost per registrant of US $209.34. Website placement predicted all four outcomes (all *P *values < .01). Yahoo! en Español yielded the highest click-through rate (0.167%) and number of registrants (n = 267). The message framing and cultural targeting interaction was not significant. Contrary to hypotheses, loss-framed ads yielded a higher click-through rate than gain-framed ads (point estimate = 1.08, 95% CI 1.03 1.14, *P = *0.004), and surface-targeted ads outperformed deep-targeted ads for clicks (point estimate = 1.20, 95% CI 1.13 1.28, *P < *.001), click-through rate (point estimate = 1.22, 95% CI 1.16 1.29, *P < *.001), and number of registrants (point estimate = 2.73, 95% CI 2.14 3.48, *P < *.001).

**Conclusions:**

Online advertising can be an effective and cost-efficient strategy to reach and engage Spanish-speaking Latino smokers in an evidence-based Internet cessation program. Cultural targeting and smoking-relevant images may be important factors for banner ad design. Online advertising holds potential for Web-based cessation program implementation and research.

## Introduction

Cigarette smoking is a major cause of disease and death in Latinos [1]. Approximately 12.5% of Latinos currently smoke [2] translating into roughly 6.3 million Latino smokers. With a projected growth of the Latino population to 133 million by 2050 (30.2% of the US population) [3,4] and the potential for smoking prevalence to increase as Latinos become more acculturated [5,6], there may be more than 16 million Latino smokers by 2050 if smoking rates remain unchecked. Tobacco cessation among Latinos is a public health priority [7,8].

Web-based cessation programs are a promising approach to reach Latino smokers with evidence-based cessation treatment. More than 60% of Latinos (32 million) are online—projected to increase to 70% by 2014 [9]—and almost half of online Latinos (45%) have used the Internet to search for health information [10]. A growing body of evidence supports the reach, efficacy, and cost-efficiency of Web-based cessation programs [11-19], but additional research is needed to understand their impact among subgroups of online smokers, including racial/ethnic minorities. In general, there are few studies of smoking cessation interventions developed specifically for Latinos [20,21], and only two studies of the effectiveness of Web-based interventions for Latinos [12,22]. One of the challenges in conducting Web-based cessation research with Latinos in the United States is that they have not engaged in Internet cessation programs as readily as other racial/ethnic subgroups [23] and recruitment of Latinos into Internet cessation trials has been challenging [11,12,22,24].

Online advertising is widely acknowledged as an increasingly important method to reach Latinos who use the Internet [25-28]. Also known as “display ads,” banner advertisements appear as graphical ads embedded into a webpage, typically including a combination of static/animated images, text, and/or video designed to convey a marketing message and/or cause the user to take an action [29]. Unlike “offline” ads (eg, billboards, newspapers, and flyers), banner ads can immediately link smokers to Web-based cessation programs, thus capitalizing on the motivation to quit when it occurs which can be critical to engaging consumers with cessation treatment [23,30]. Moreover, banner ads can target consumers by strategic placement on selected websites with synergistic content, known demographic profiles, or past online behavior. Therefore, online advertising may represent a “participant-friendly” and cost-efficient solution to reach and recruit online Latino smokers to Web-based cessation programs [31-34].

Reaching online Latino smokers requires not only efficient and effective advertising channels, but also an understanding of the target audiences’ preferred content and context of messages [32,33,35]. Approximately half of online Latinos use the Internet either primarily in Spanish (19%) or in both English and Spanish (28%), with the growth of both segments outpacing English language usage as more Spanish content becomes available online [36]. Therefore, we tested ads in Spanish to reach this large and growing segment of online Latinos. With regard to message content, Prospect Theory [37,38] suggests that people respond differently to factually equivalent messages depending on whether they are framed to emphasize benefits through gain-framed messages (eg, “if you quit smoking, you will live longer”) or costs through loss-framed messages (eg, “if you do not quit smoking, you will die sooner”). Applied to health behaviors, research suggests that when behaviors have a relatively certain outcome, gain-framed messages are more persuasive; if behaviors result in an uncertain outcome, loss-framed messages are more effective [39]. Because quitting smoking will almost certainly prevent disease, Prospect Theory predicts that gain-framed messages will be more persuasive than loss-framed messages. Recent evidence suggests that highlighting the benefits of quitting (through gain-framed messages) is more effective to encourage preventive behaviors such as smoking cessation [40-42]. However, there have been few studies that have examined the impact of message framing in health interventions designed specifically for racial/ethnic minorities [43,44].

With regard to message context, research with Latinos indicates that addressing cultural elements is critical in developing smoking cessation interventions [5,45-48]. The process of designing messages around group-level characteristics has been referred to as both cultural tailoring and cultural targeting [49-50]. We use the term “cultural targeting” in this study to refer to the delineation of a particular population segment. Cultural targeting can be done at a surface level (eg, attending to the visual characteristics or language of intervention materials) or at a deep level (eg, incorporating specific Latino cultural values) [51]. In general, few studies have investigated how surface- and deep-targeted messaging functions in specific subgroups of racial/ethnic minorities [52].

The purpose of this study was: (1) to investigate whether online advertising is an effective strategy to promote engagement with a Spanish-language cessation website among online Latino smokers, and (2) to identify the optimal message characteristics for this audience. To address these questions, we conducted a two-phased, mixed-methods study. In Phase I, formative research with online Latino smokers guided the development of four Spanish-language banner ad prototypes (deep-targeted/gain-framed, deep-targeted/loss-framed, surface-targeted/gain-framed, and surface-targeted/loss-framed). In Phase II, we tested the effectiveness of these four ads in reaching and recruiting Latino smokers to a free Spanish-language smoking cessation website. The primary outcomes in Phase II were: (1) the absolute number of clicks on an ad, (2) the click-through rate to the smoking cessation website, (3) the number of ad responders who went on to register on the cessation website (“registrants”), and (4) the cost per registrant. We hypothesized there would be an interaction between message framing and cultural targeting, such that the deep-targeted/gain-framed ad would outperform all others on all metrics. Based on Prospect Theory, we also hypothesized that gain-framed messages would outperform loss-framed messages on all metrics holding constant cultural targeting. Finally, we hypothesized that deep-targeted ads would outperform surface-targeted ads on all metrics holding constant the message frame.

## Methods

### Phase I: Advertising Development and Pre-testing

**Figure 1:**
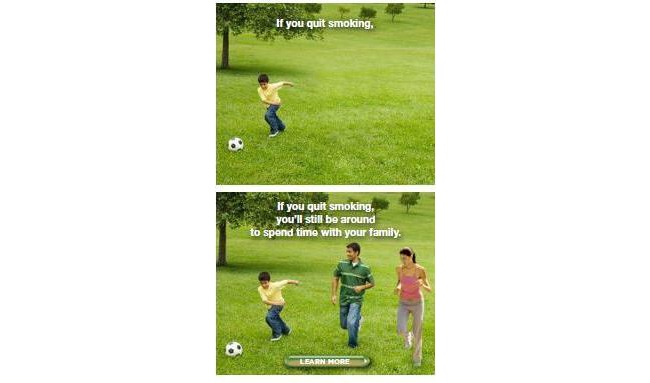
Gain-framed/deep-targeted ad for familismo.

A multicultural, multidisciplinary, and bilingual expert panel with expertise in behavioral science, marketing, communication technology, and advertising identified two dominant cultural values to consider for cultural targeting of messages to Latino smokers: *familismo *and *fatalismo*. *Familismo *is central to the Latino health experience; family and close friends are often consulted for general health issues before a medical professional, and family is often a strong motivator of health behavior. *Fatalismo *refers to the belief “*si Dios quiere*” (“what God wants”) which often guides Latino perceptions about their control over health and wellness. Our formative Phase I focused on developing banner ad prototypes for each cultural value that crossed message frame (gain vs loss) with level of cultural targeting (surface vs deep) resulting in a total of eight Spanish-language ads (see Figure 1). All ads were written in Spanish, developed in Adobe Flash with animation and motion by a professional creative developer, and included a call to action (ie, “click here for more information”).

To examine preferences for ad characteristics, 200 American Spanish-speaking smokers were recruited from two Spanish-language websites: MSN Latino [53] and MySpace Latino [54]. A study invitation banner ad transferred potential participants to an online screening form where eligible individuals (ie, current smokers, aged 18 or older, and Hispanic/Latino ethnicity of any race) provided online consent and completed a survey. Survey items included demographics, smoking history, Internet use, general receptivity to online ads, and the language subscale of the Short Acculturation Scale for Hispanics (SASH) [55]. Next, each participant sequentially viewed two of the eight ads. The two ads differed on cultural value (*familismo *vs *fatalismo*), but were identical in message frame and depth of cultural targeting (eg, gain-framed/deep-targeted) for direct comparison of message characteristics. Ad presentation was counterbalanced so that an equal number of participants saw each of the 8 ads first. Participants were asked to indicate their receptivity to the ads using a 10-point Likert scale (1 = not at all, 10 = very much). Participants also responded to 12 statements that addressed the strength of cultural targeting (eg, this ad is for someone like me), ad credibility (eg, this ad is believable), perceived efficacy of the ad (eg, this ad has me thinking about quitting smoking), and intention to respond (eg, I would click on this ad if I saw it) using a 5-point Likert scale (1 = strongly disagree, 5 = strongly agree). Finally, both ads were presented in tandem, and participants were asked which one they liked the most and why. An equal number of participants viewed each ad pair. The survey was administered in Spanish.

#### Data Analysis

Descriptive statistics were used to examine the demographic, smoking history, Internet use, online advertising receptivity, acculturation measures, and ratings of individual ad characteristics. Analysis of variance (ANOVA) procedures, Chi-square tests (χ^2^), and *t *tests were used to evaluate differences between the two ad types (*familismo *vs *fatalismo*).

### Phase II: Evaluation of Online Banner Ad Effectiveness

Phase II examined the effectiveness of the four final ads (see Multimedia Appendix) in promoting the free Spanish-language version of BecomeAnEX (www.becomeanex.org) [56]. All ads were tested in Spanish (the English language version of each ad is shown in the Appendix for reference only). BecomeAnEX is a free, branded smoking cessation website designed to engage smokers through videos, interactive content, a personalized quit plan, and an online community of current and former smokers. Using a Latin square design (Table 1), the four ads (deep-targeted/gain-framed, deep-targeted/loss-framed, surface-targeted/gain-framed, and surface-targeted/loss-framed) were systematically rotated across four Spanish-language websites over four time periods of approximately one month each between August and November 2009. This design controlled for variability in ad performance associated with website placement and time.

The four websites chosen for this study were MySpace Latino [54], MSN Latino [53], MiGente [57], and Yahoo! en Español [58]. Our media partner, PHD Media LLC, examined various metrics of reach and performance [59] using data from comScore [60]—a widely used Internet analytics company—to identify sites with the best ability to reach our target audience of American adult Latinos. We selected a mix of portals with vast foreign-language segments and broad reach to Latino users as well as social network sites that specifically targeted Latinos. Banner ads ran on the home page and in various sections of each site (eg, email, weather, news, women’s health, and men’s health) to optimize their reach.

Banner ads were assigned a click tag that linked an individual’s use of the BecomeAnEX website with the specific banner ad that prompted the visit. Each time an individual visited a website where one of the banner ads was placed, an impression (a unit of analysis analogous to a page view) was recorded by a tracking software program. If the user clicked on the banner ad, a click-through was also recorded. Click-through rate is calculated by dividing the total number of clicks by the total number of impressions, expressed as a percentage. This is a standardized metric that allows for comparison of ad performance across websites and varying costs by essentially controlling for the number of impressions [61]. All banner ads linked directly to the Spanish BecomeAnEX home page. When a user arrived at the BecomeAnEX website from a banner ad, a “home page visit” was recorded and the click tag associated with the banner ad was recorded along with standard website utilization metrics (eg, whether the individual registered to become a member, number of page views, and number of minutes online). Advertising media contracts with the four websites were designed to ensure a specific number of impressions of each banner ad over each of the four time blocks. Advertising costs by website for each time block are shown in Table 1. We also report the cost per click for each ad—calculated as the total cost divided by the number of clicks in a given ad campaign—to enable comparison to other published studies. Cost per registrant is calculated as the total cost divided by total number of registrants in a given ad campaign.

**Table 1:** Overview of Latin square design and overall banner ad performance by website placement and time.

Website	Metric	Month 1	Month 2	Month 3	Month 4
MySpace Latino	Ad type	Surface gain	Deep loss	Deep gain	Surface loss
	Cost (US$)	$5100.00	$5100.00	$5100.00	$5100.00
	Impressions	1,517,216	1,520,018	1,519,437	1,520,487
	Clicks	1146	988	1080	1278
	Click-through rate^a^	0.076%	0.065%	0.071%	0.084%
	Registrants	19	4	4	7
	Cost per click^b ^(US$)	$4.45	$5.16	$4.72	$3.99
	Cost per registrant^c ^(US$)	$268.42	$1275.00	$1275.00	$728.57
Yahoo	Ad type	Surface loss	Surface gain	Deep loss	Deep gain
	Cost (US$)	$8040.00	$8016.52	$7997.56	$7972.60
	Impressions	1,342,521	1,336,274	1,332,926	1,328,766
	Clicks	2737	2388	2052	1741
	Click-through rate	0.204%	0.179%	0.154%	0.131%
	Registrants	109	101	34	23
	Cost per click (US$)	$2.94	$3.36	$3.90	$4.58
	Cost per registrant (US$)	$73.76	$79.37	$235.22	$346.63
MSN Latino	Ad type	Deep gain	Surface loss	Surface gain	Deep loss
	Cost (US$)	$6827.00	$6791.74	$6852.86	$6771.21
	Impressions	1,498,058	1,490,065	1,509,246	1,489,548
	Clicks	1776	2289	2396	2185
	Click-through rate	0.119%	0.154%	0.159%	0.147%
	Registrants	37	71	74	11
	Cost per click (US$)	$3.84	$2.97	$2.86	$3.10
	Cost per registrant (US$)	$184.51	$95.66	$92.61	$615.56
Mi Gente	Ad type	Deep loss	Deep gain	Surface loss	Surface gain
	Cost (US$)	$6250.00	$6250.00	$6250.00	$6250.00
	Impressions	1,745,792	2,116,132	1,781,070	1,781,451
	Clicks	653	673	703	737
	Click-through rate	0.037%	0.032%	0.039%	0.041%
	Registrants	0	1	3	2
	Cost per click (US$)	$9.57	$9.29	$8.89	$8.48
	Cost per registrant (US$)	N/A^d^	$6250.00	$2083.33	$3125.00

^a ^the number clicks divided by the number of impressions

^b ^cost divided by the number of clicks in a given ad campaign (not an outcome metric in this study, but included in the table for comparison to other published studies)

^c ^cost divided by total number of registrants in a given ad campaign

^d ^cost per registrant cannot be calculated since there were 0 registrants during this segment of the study

#### Data Analysis

First, descriptive statistics were calculated for each of the four outcome variables (ie, absolute number of clicks, click-through rate, number of registrants, and cost per registrant) by website placement, time, and ad type. Next, a series of regression models explored the interaction of message framing and cultural targeting, and the main effects of website placement, time, message framing, and cultural targeting. Three of the outcome variables are count data (eg, clicks and registrants) or are based on count data (eg, click-through rate) and typically would be analyzed using Poisson regression. However, since the variance of these outcomes demonstrated over-dispersion, we determined that Poisson regression models would not be a good fit. Instead, we used negative binomial regression models to examine clicks, registrants, and click-through rate, and general linear regression models to examine cost per registrant. We evaluated the presence of an interaction between message framing and cultural targeting by including an interaction term in the model. For those models without evidence of an interaction, we evaluated separately the main effects of message framing and cultural targeting for loss-framed versus gain-framed ads and surface-targeted versus deep-targeted ads, respectively. All statistical analyses were performed using SAS Version 9.2 for Windows [62].

## Results

### Phase I: Prototype Development and Pre-testing

A total of 5,698,776 impressions to the Phase I recruitment banner ad were generated from MSN Latino and MySpace Latino between June 1-24, 2009. A total of 8231 individuals clicked on the study invitation ad. Of those who reached the survey site, approximately half (3712/7436, 49.92%) completed eligibility screening and 25.51% (947/3712) met the eligibility criteria. Of those eligible, 67.2% (636/947) provided informed consent, and of those who consented, 32.1% (204/636) completed the survey.

A total of 204 Latino current smokers completed the survey: 56.4% (115/204) were male, average age was 36.7 years (SD 11.7, range 18-70), 53.9% (110/204) were white, 3.4% (7/204) black, 4.9% (10/204) American Indian/Alaskan native, 1.5% (3/204) native Hawaiian/other Pacific Islander, 0.5% (1/204) Asian, and 35.8% (73/204) choose the “other” category. Participants used the “other” category to describe multiracial background and country of origin. The mean score on the SASH was 1.63 (SD 0.56, range 1.0-3.5) indicating a low level of acculturation. Respondents reported smoking an average of 10.1 cigarettes per day (SD 9.7). Over half (56.9%, 116/204) reported smoking every day (43%, 88/204 smoke some days). The average number of smoking days per month was 22.9 (SD 9.7). Approximately half (48.5%, 99/204) had their first cigarette > 60 minutes after waking suggesting a low level of nicotine dependence, and 84.3% (172/204) expressed a desire to quit smoking within the next 6 months. Respondents were largely Internet-savvy and receptive to online advertising: 72.5% (148/204) reported using the Internet several times a day and about half (49.5%, 101/204) reported using the Internet for more than 5 years. Two-thirds (66.2%, 135/204) of respondents viewed Web advertising favorably (ie, like it “somewhat” or “a lot”).

All eight ads were reviewed positively, with mean ratings of “liking” ranging from 7.24 (SD 2.8) to 8.17 (SD 2.6). For the 12 items related to ad characteristics, there were no differences between ratings of *familismo *versus *fatalismo *ads: all eight ads were rated positively across all 12 dimensions both with regard to mean score and proportion of positive responses. When presented with both ads simultaneously, most of the participants (55.9%, 114/204) preferred the *familismo *ad, and gave reasons that related directly to its cultural relevance and the value of *familismo *(eg, “Both talk about quitting smoking, but this one tells me to think about my family;” “This ad definitely has a more profound message and most of all talks about family—it is more profound and I identify with it;” or “Because it speaks of future consequences that could happen; both [ads] are important but I believe the family one leaves more of a mark”). Given these results, we selected the Latino cultural value of *familismo*—illustrated by a young boy playing soccer with his parents—to incorporate into the deep-targeted ads evaluated in Phase II.

### Phase II: Evaluation of Online Banner Ad Effectiveness

Across the Phase II study period (August 10 to November 29, 2009), a total of 24,829,007 impressions were placed across the four websites for the four Spanish-language banner ads. There were 24,822 clicks on the banner ads, yielding an overall click-through rate of 0.10%. A total of 500 unique individuals registered on BecomeAnEX, yielding a conversion rate of 2.77%. The cost of the online advertising efforts totaled US $104,669.49, resulting in an overall cost per click of US $4.22 and an overall cost per registrant of US $209.34.

As shown in Table 2, website placement was a significant predictor of clicks (point estimate = 0.51, 95% CI 0.46 0.56, *P < *.001), click-through rate (point estimate = 0.45, 95% CI 0.41 0.48, *P < *.001), number of registrants (point estimate = 0.14, 95% CI 0.10 0.20, *P < *.001), and cost per registrant (*P = *0.01). The MiGente website yielded the lowest number of clicks (n = 2766), click-through rate (0.037%), and number of registrants (n = 6), and highest cost per registrant (US $4166.67). Yahoo! and MSN Latino were the two best performing websites, with Yahoo! yielding a higher click-through rate (0.167% vs 0.144%; χ^2^_1 _= 9.8, *P = *.002) and higher number of registrants (267 vs 193; χ^2^_1 _= 8.0, *P *= .005).

Our main hypothesis that there would be a significant interaction between message framing and cultural targeting was not supported by any of the four outcomes (all *P *values > .05). Descriptive data for the message framing and cultural targeting interaction are shown in Table 3. Our second hypothesis that gain-framed ads would outperform loss-framed ads holding constant level of cultural targeting was not supported for absolute number of clicks, the number of registrants, or the cost per registrant. There was a statistically significant difference in click-through rate, with loss-framed ads yielding a higher click-through rate than gain-framed ads (0.105% vs 0.095%, point estimate = 1.08, 95% CI 1.03 1.14, *P *=.004). Our third hypothesis was that deep-targeted ads would outperform surface-targeted ads holding message frame constant; however, the results showed that for all outcomes, surface-targeted ads performed better than deep-targeted ads. Surface-targeted ads outperformed deep targeted ads on clicks (13,674 vs 11,148; point estimate = 1.20, 95% CI 1.13 1.28, *P < *.001), click-through rate (0.111% vs 0.089%; point estimate = 1.22, 95% CI 1.16 1.29, *P < *.001), registrants (386 vs 114; point estimate = 2.73, 95% CI 2.14 3.48, *P < *.001), and cost per registrant (US $135.75 vs US $458.49, *P < *.001).

**Table 2:** Banner advertising results by website placement.

Metric	Website				*P *value
	Yahoo! en Español	MSN Latino	MySpace Latino	MiGente	
Clicks	8918	8646	4492	2766	< .001
Click-through rate	0.167%	0.144%	0.074%	0.037%	< .001
Registrants	267	193	34	6	< .001
Cost per registrant (US$)	$119.95	$141.15	$600.00	$4166.67	.01

**Table 3:** Banner advertising results by message framing and cultural targeting.

Message Frame	Metric	Level of cultural targeting		
		Surface	Deep	Total
Loss	Clicks	7007	5878	12,885
	Click-through rate	0.114%	0.097%	0.105%
	Registrants	190	49	239
	Cost per registrant (US$)	$137.80	$533.04	$218.83
Gain	Clicks	6667	5270	11,937
	Click-through rate	0.109%	0.082%	0.095%
	Registrants	196	65	261
	Cost per registrant (US$)	$133.77	$402.30	$200.65
Totals	Clicks	13,674	11,148	24,822
	Click-through Rate	0.111%	0.089%	0.100%
	Registrants	386	114	500
	Cost per registrant (US$)	$135.75	$458.49	$209.34

## Discussion

This is one of the first studies to examine the impact and costs of online Spanish-language banner advertising to reach and recruit Latino smokers to a Web-based cessation intervention. Our strategy was specifically designed to test the efficacy of various messaging elements and website placement to generate online smoker response and engagement. Overall, the results demonstrated that online advertising can effectively reach Latino smokers: during the 4-month study period, 24,822 individuals responded to banner advertising yielding a click-through rate of 0.10%; of these, 500 registered on the BecomeAnEX Spanish-language smoking cessation website.

The overall click-through rate in this study (0.10%) compares favorably to industry averages [63] and other research studies. Klausner et al [64] used online banner ads to promote a San Francisco Department of Public Health website for syphilis with an overall click-through rate in the 2-month study of 0.1% with different ads yielding click-through rates of 0.05% to 0.14%. Bull et al [65] evaluated the effectiveness of online banner ads for recruitment to human immunodeficiency virus (HIV) and sexually transmitted disease (STD) prevention research trials and reported a click-through rate of 0.05%. A recent study examining the effectiveness of online study recruitment via Facebook advertising yielded a click-through rate of 0.05% [66]. To some, these rates may raise questions about the generalizability of banner advertising. However, it is important to remember that of the tens of thousands of impressions placed on any given website, only a small percentage of those who view a banner ad may be members of the target audience or meet eligibility criteria. Given the current and growing magnitude of the Latino online audience, even reaching a small percentage can translate into relatively large numbers of smokers. This recruitment yield may, in fact, be comparable to or even higher than a newspaper or radio advertisement that yields several hundred responses when thousands of individuals may have been exposed to the ad. The primary difference is that the denominator can be determined with more certainty in online advertising compared to traditional mass media [30].

Results also indicate that online banner ads can be cost efficient, particularly when compared to other traditional program recruitment methods for racial/ethnic minority participants. While there was significant variation in cost per registrant across website placement and message effects, the most efficient and cost-effective advertising approach (surface-targeted ads placed on Yahoo! en Español) yielded 210 registrants in 2 months at a cost of US $73-$79 per registrant. These results compare favorably to other efforts to recruit minority participants using traditional approaches (ie, proactive face-to-face methods or reactive “offline” media-based methods) that reported lower and/or slower recruitment yield at higher cost [67-69]. The capacity of online advertising to reach and recruit 500 Latino smokers to a cessation website during a 4-month period has important implications for cessation-related eHealth research. Registered users of cessation websites report high levels of motivation to quit [13,24,70-74] and may represent the strongest pool available to efficiently recruit Latino smokers into clinical trials.

Contrary to the published literature and our hypotheses, results indicated that surface targeting was a more effective approach than deep targeting. However, these results should be considered within the context of several study parameters. The use of family imagery to reflect deep-level targeting and tobacco product imagery to reflect surface-level targeting was intentional because we were interested in examining two different types of imagery. It is possible that surface-targeted ads may have yielded better results because smokers are more likely to attend and respond to images featuring tobacco products given their more obvious relevance. Thus, we cannot conclude from this study that deep-level targeting is not effective. Future research should consider an additive approach in which deep cultural targeting graphics include culturally relevant elements as well as tobacco products/images to catch the attention of smokers.

Also contrary to our hypotheses, loss-framed messages yielded a higher click-through rate than gain-framed messages. This finding is intriguing, especially in light of the non-significant results for the other outcome metrics for message framing. On the one hand, it suggests that the behavior of clicking on an ad is indeed sensitive to message frame whereas the more distal behavior of registering on a cessation website is not. However, a number of variables may explain why this may be true. For example, previous advertising research has shown that consumers’ level of involvement or interest with a product or issue exerts a strong influence on the effect of message framing [75]. Consumers with high involvement pay more attention to the issue of losses, whereas consumers with low levels of involvement with a product or issue pay more attention to gains in messaging [76]. Thus, response variation across websites may reflect varying levels of engagement whereby visitors who are specifically looking to obtain general news information, check email, or connect in an online social network were more likely to attend to an ad with negatively framed content. Future research will be important to examine factors such as consumer involvement, ad placement, and message processing to better understand how to engage smokers at the point of an initial recruitment message.

Data from this study also suggest that social media websites may not be as fruitful for online recruitment as more general websites. Click-through rates from the MiGente and MySpace websites were significantly lower than MSN Latino and Yahoo! en Español. Bull and colleagues [77] also found lower-than-average click-through rates for MiGente (0.06%). These lower click-through rates may reflect differences in engagement patterns on social networking sites as compared to websites related to news and information, particularly for Latinos. Social networking sites, which seek to maximize interpersonal connections, may elicit high levels of engagement and possibly lower the probability of attending to an advertisement or of having a positive attitude toward online advertising on the site. An exception to this hypothesis is Facebook which has been an effective context for research recruitment [66,78] perhaps due to its particular advertising guidelines. Future research is needed to understand the opportunities and challenges of using online social networks to reach and recruit smokers to cessation interventions [79].

The results should be considered within the context of several limitations. First, campaign costs reflect a variety of factors and may be difficult to replicate. Specific information related to costs and advertising yield is provided to enable comparisons. Second, the extent to which our results are generalizable to other websites is unclear. The similarity in results obtained for Yahoo! en Español and MSN Latino lends support to the notion that other portals may perform similarly, but this remains to be tested. Relatedly, these findings are limited to Latinos using a Spanish-language website. The effectiveness of online ads in reaching English-preferring or bilingual Latinos should be addressed in future research. Third, it is possible that repeat visitors to any of the four websites where we placed banner ads could have seen more than one study-related ad, and this repeat exposure may have primed a subsequent click. Fourth, although not specifically a limitation, it is important to note that each of the four outcomes may be influenced by various factors, including but not limited to the variables examined in this study (ie, message framing and cultural targeting). For example, click-through rate is largely determined by the content of an ad, but it may also be a function of the degree to which users of certain websites attend to advertising; the number of registrants involves not only clicking on an ad but also then perusing a website and deciding to provide personal information to register. Lastly, the reach of this method is obviously limited to those Latino smokers who are online. Alternative strategies are needed to reach “offline” Latino smokers with cessation services.

Despite these limitations and considerations, this study has a number of strengths. Evaluating the use of online advertising to promote evidence-based online cessation treatment among Spanish-speaking Latino smokers is unique within the field of tobacco control. We assembled a multicultural, multidisciplinary, and bilingual team with expertise that spans behavioral science, marketing, communication technology, and advertising to examine how to optimize our ability to reach and recruit online Latino smokers to an evidence-based cessation program. The Latin square quasi-experimental design was extremely efficient in allowing us to control for two potentially confounding factors: the volume of website traffic and time. We considered other research designs and approaches including laboratory experiments to examine cognitive processing factors such as message recall, message recognition, and psychological variables that mediate or moderate response to ads. However, message testing under highly controlled conditions at the expense of external validity would not yield the kind of information needed to successfully promote registration to a Web-based cessation website that could be used to inform recruitment of Latino smokers in subsequent cessation trials. We encourage the use of quasi-experimental methods as appropriate and hope that the results presented herein provide a useful benchmark in future eHealth research. Our approach may also help other public health researchers consider how to use the Internet for disease prevention.

In summary, identifying the effectiveness of various advertising parameters to promote an evidence-based online cessation program among Spanish-speaking Latino smokers holds potential for online program implementation and intervention research. Although a large number of Latino smokers use the Internet and are interested in using it for smoking cessation, the participation rate of Latinos in Web-based cessation interventions has been relatively low. Digital metrics such as click-through rate and registration rate related to various online message characteristics provide a deeper understanding of factors related to actual attention and appeal of online programs, particularly for specific subgroups [80]. Future research is needed to build on the methods used in this preliminary investigation to better understand how to increase consumer demand for tobacco cessation services, especially among racial/ethnic minorities [81].

## Multimedia Appendix 1

Final banner ads in English (N=4) and Spanish (N=4).

## Abbreviations

ANOVA: Analysis of variance
SASH: Short Acculturation Scale for Hispanics
