# Supplementary material for: Online Advertising to Reach and Recruit Latino Smokers to an Internet Cessation Program: Impact and Costs
Source: J Med Internet Res. 2012 Aug 27;14(4):e116. doi: 10.2196/jmir.2162 (PMC3510691; doi:10.2196/jmir.2162)

Multimedia Appendix

Deep-Targeted / Gain-Framed banner ad, English language version

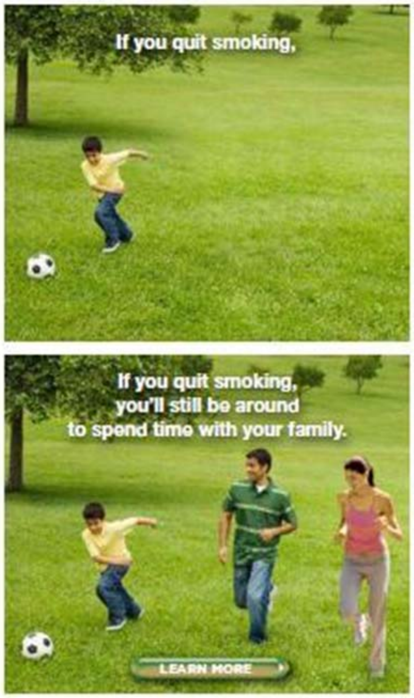

Deep-Targeted / Gain-Framed banner ad, Spanish language version

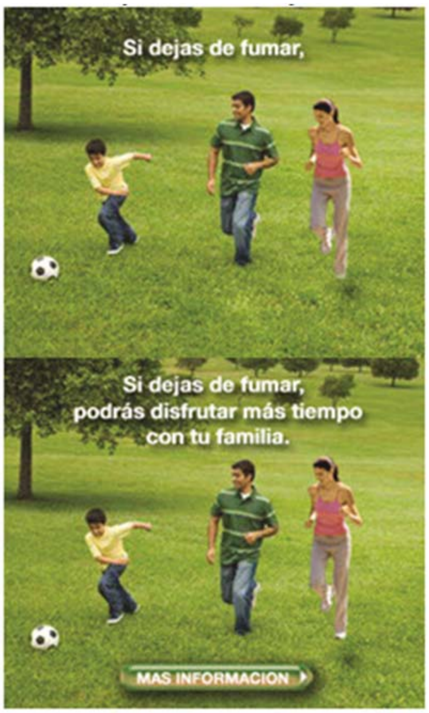

Deep-Targeted / Loss-Framed banner ad, English language version

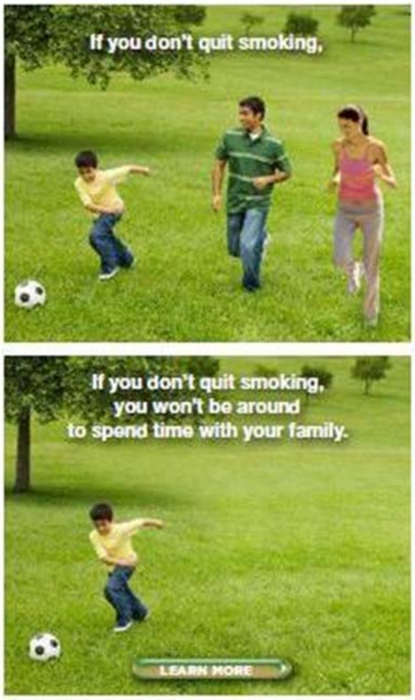

Deep-Targeted / Loss-Framed banner ad, Spanish language version

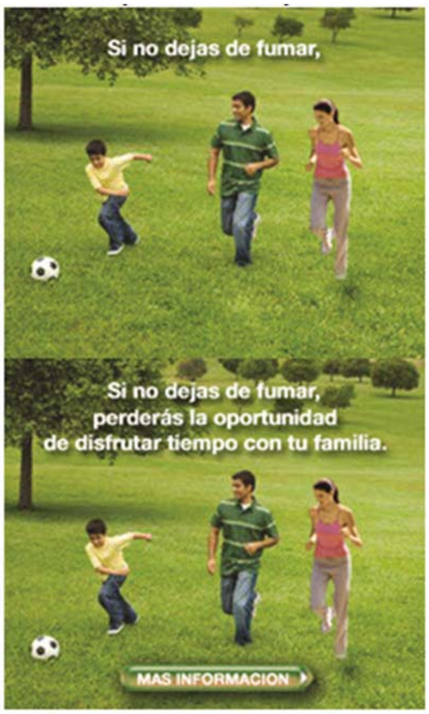

Surface-Targeted / Gain-Framed banner ad, English language version

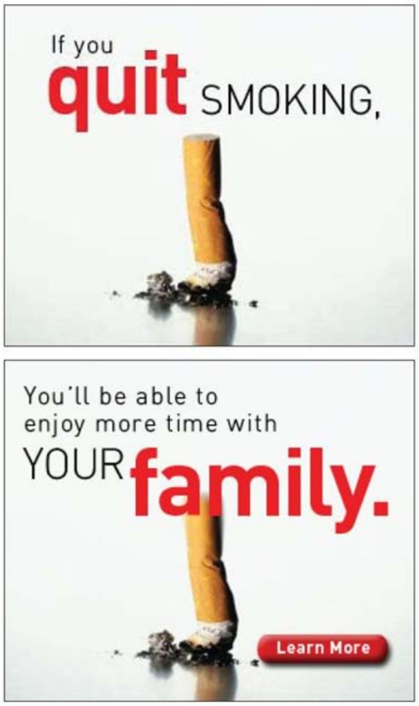

Surface-Targeted / Gain-Framed banner ad, Spanish language version

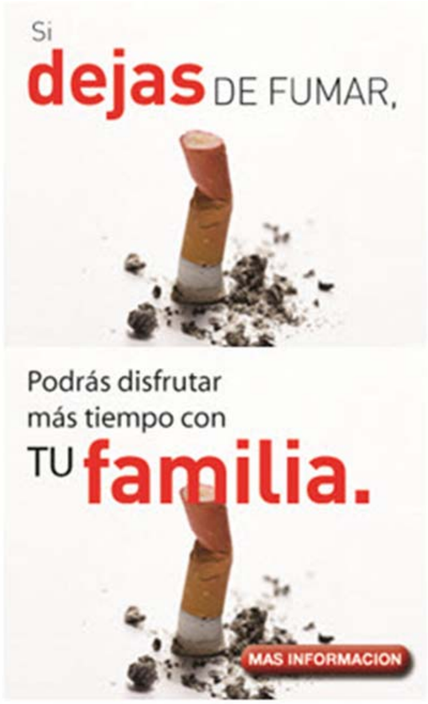

Surface-Targeted / Loss-Framed banner ad, English language version

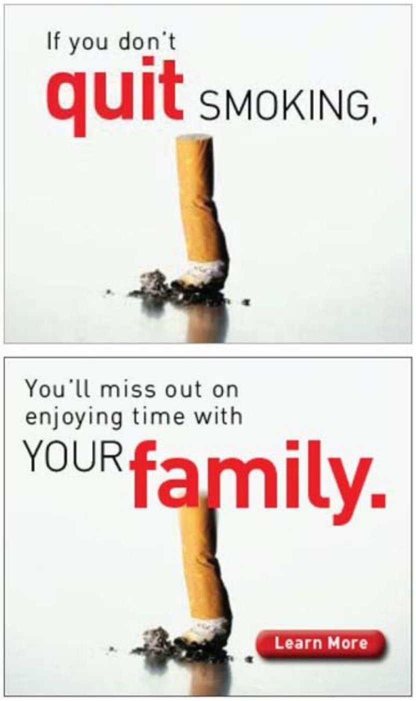

Surface-Targeted / Loss-Framed banner ad, Spanish language version

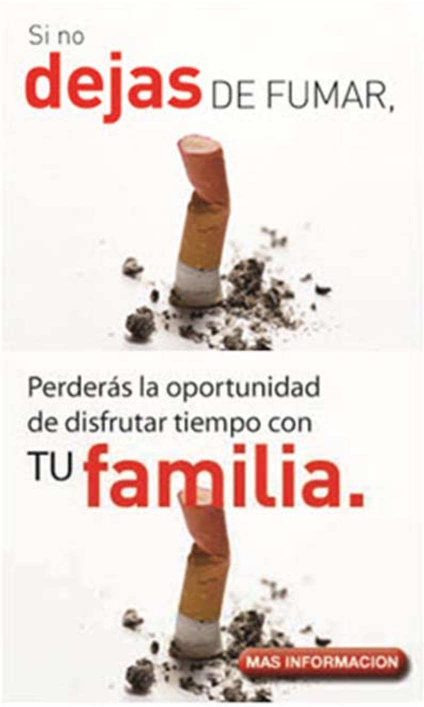

Supplement: Supplementary file 1 [file jmir_v14i4e116_app1.pdf]
